# A principal component entropy metric for assessing global synchronicity in EEG signals

**DOI:** 10.1038/s41598-026-36434-0

**Published:** 2026-03-03

**Authors:** Luis Diambra, Anna Hutber, Zakarriah Drakeford-Hafeez, Ran Mi, Vasiliki Tsirka, Alberto Capurro

**Affiliations:** 1 Blizard Institute, Queen Mary University of London, London, UK; 2 CENEXA, UNLP, La Plata, Argentina; 3 CCT-La Plata, CONICET, La Plata, Argentina; 4 Department of Neurosciences, Royal London Hospital, Barts Health NHS Trust, London, UK

**Keywords:** EEG signals, Synchronisation, Functional connectivity, PCA, Entropy, Computational biology and bioinformatics, Neuroscience

## Abstract

Neuronal oscillations and their inter-areal synchronisation are fundamental for brain function and cognitive processes. While electrophysiological recordings, such as electroencephalography (EEG), provide invaluable insights, existing quantitative methodologies for assessing neuronal synchrony in EEG often focus on pairwise interactions, thereby limiting a comprehensive understanding of global network coordination. This study proposes principal component (PC)-entropy, a novel multichannel synchronisation metric designed to quantify the global degree of synchrony within brain signals. PC-entropy is a hybrid measure derived from Principal Component Analysis and Shannon entropy, specifically by applying normalised entropy to the eigenvalues obtained from data covariance. This approach effectively translates the distribution of variance across principal components into a synchrony measure, ranging from 0 (perfect synchrony) to 1 (complete desynchronisation), and is notably robust to variations in the number of recording channels. We validated PC-entropy using synthetic data from the Kuramoto model, including non-isofrequency signals, demonstrating its efficacy in assessing synchronisation. PC-entropy was then applied to three human EEG datasets, demonstrating its utility in detecting neural synchrony changes during sleep, differentiating nocturnal frontal lobe epilepsy (NFLE) patients from controls, reflecting consciousness levels in coma patients, and distinguishing arithmetic task performance. PC-entropy offers a valuable and sensitive tool for assessing global brain synchrony. It provides a new dimension for understanding functional connectivity and various physiological states, extending beyond the limitations of pairwise analyses and conventional spectral approaches.

## Introduction

Cognitive processes emerge from the coordinated exchange of information among functionally specialised brain regions. Neuronal oscillations provide a fundamental mechanism for this dynamic coordination, arising from synchronised activity within local neuronal ensembles^1–4^. The brain dynamically orchestrates information flow between these neural nodes according to task demands and behavioural state, with communication patterns reflected in the amplitude, frequency, and phase relationships of oscillatory activity across the network^5^.

The notion that neuronal oscillations and their inter-areal synchronisation play a vital role in normal brain function has spurred the development and application of quantitative methodologies for assessing neuronal synchrony in electrophysiological recordings over the last few years^7^. Electroencephalography (EEG) and magnetoencephalography (MEG) currently provide the primary non-invasive approaches for directly recording electrical brain activity across different regions^8^. While EEG is widely employed for monitoring brain activity, clinical interpretation relies heavily on visual inspection by trained experts, often supplemented by signal processing techniques. This subjective approach presents inherent limitations, as interpretations may be prone to inter-rater variability and potential bias^9^.

EEG signals enable quantitative assessment of inter-regional synchronisation, statistical dependencies and spectral characteristics across brain networks. Measuring inter-regional neuronal synchrony has been critical for characterising network dynamics in physiological and pathological brain states across frequency bands that reflect distinct oscillatory mechanisms. Numerous methods have been developed to measure synchrony in electrophysiological data^10^, including coherence, cross-correlation^11^, mutual information^12^, partial directed coherence^13^, phase-locking value^14^, and Granger causality^15^. These predominantly frequency-domain approaches are readily accessible through established open-source toolboxes^16,17^, with their respective strengths and limitations thoroughly characterised in the literature^18^. However, these approaches are predominantly pairwise measures that cannot capture the global synchronisation state across multiple channels simultaneously^19^. More recently, two important extensions of pairwise approaches have been applied to assess global synchrony. One such method, global phase synchronisation, determined that REM sleep exhibits a greater degree of synchrony than NREM sleep^6^. The second extension, global field synchronisation, a wavelet transform-based method, has been used to detect a marked reduction in theta and alpha connectivity in the frontal regions of the brain of children with autism spectrum disorder^20^.

In this paper, we introduce PC-entropy, a novel multi-channel synchronisation metric that quantifies global network synchrony by computing Shannon entropy over the normalised eigenvalues of the covariance matrix. This approach combines two established analytical tools, principal component analysis and Shannon entropy, that have been extensively applied to EEG analysis, but not previously integrated for inter-channel synchronisation assessment. We validate PC-entropy using coupled phase oscillator systems (Kuramoto model) and demonstrate its application across multiple EEG paradigms, revealing new insights into functional connectivity patterns. We first detail the computational approach and describe the synthetic and experimental datasets used for validation, then present performance on non-isofrequency Kuramoto model simulations and diverse EEG recordings. Finally, we evaluate the method’s utility for global synchrony assessment and its implications for understanding network dynamics.

## Methods

### Eigenvalues and PC-entropy

The proposed method integrates Principal Component Analysis (PCA) with Shannon entropy to create a mathematically robust synchronisation measure. PCA involves eigen-decomposition of the covariance matrix^21^. Let the real-valued data matrix $\textbf{X}$ be of $N\times M$ size, where *N* is the number of sample points and *M* is the number of variables. The covariance matrix $\textrm{Cov}_X$ of *X* is given by:

1 $$\begin{aligned} \textrm{Cov}_\textrm{X} = \frac{1}{\textrm{N}-1} (\textbf{X}-\bar{\textbf{X}})^\textrm{t} (\textbf{X}-\bar{\textbf{X}}), \end{aligned}$$

where the transpose of the matrix is denoted by using the superscript $\textrm{t}$ in the given matrix. $\textrm{Cov}_\textrm{X}$ is a symmetric matrix and so it can be diagonalised:

2 $$\begin{aligned} \textrm{Cov}_\textrm{X} = \textbf{V} \textbf{L} \textbf{V}^\textrm{t} \end{aligned}$$

where $\textbf{V}$ is a matrix of eigenvectors (each column is an eigenvector) and $\textbf{L}$ is a diagonal matrix with the eigenvalues $\lambda _i$ with $i=1,\ldots ,M$ in decreasing order on the diagonal. The eigenvectors are called the principal axes of the data. Projections of the data onto the principal axes are called principal components. Thus, they can be seen as transformed variables that capture the highest percentage of explained variance from the original dataset. Alternatively, PCA can also be performed through singular value decomposition (SVD) of the data matrix $\textbf{X}$ as follows:

3 $$\begin{aligned} \textbf{X} = \textbf{U} \textbf{S} \textbf{V}^\textrm{t}, \end{aligned}$$

where $\textbf{U}$ is a unitary matrix and $\textbf{S}$ is the diagonal matrix with singular values $s_i$. The singular values are related to the eigenvalues of the covariance matrix by $\lambda _i=s^2_i/(n-1)$. The second component assesses the redundancy of data through entropy, which quantifies uncertainty or disorder in a dataset. In information theory, entropy reflects the average uncertainty of a probability distribution $\{p_i\}$. Following Shannon^22^, entropy is defined as:

4 $$\begin{aligned} H[p_i]= -\sum _i^M p_i \ \log _2(p_i). \end{aligned}$$

This yields entropy in *bits* due to the base-2 logarithm. Entropy (4) is maximised when probabilities $\{p_i\}$ are uniformly distributed, representing maximum complexity and minimum redundancy $\textbf{X}$. In this case, maximum entropy equals $H_{max}=\log _2(M)$, depending on the number of channels. For comparing EEG recordings with different channel counts, normalisation is essential. This can be achieved when the entropy (4) is divided by the maximum entropy:

5 $$\begin{aligned} \bar{H}[p_i]= -\frac{1}{\log _2(M)}\sum _i^M p_i \ \log _2(p_i). \end{aligned}$$

This normalised entropy^23^ is base-independent, scales output range [0,1] and handles temporary channel loss in recordings.

To apply this framework to eigenvalues $\lambda _i$; which do not satisfy probability axioms, they were normalised as follows:

6 $$\begin{aligned} \bar{\lambda _i}= \frac{\lambda _i}{\sum _i^M \lambda _i}. \end{aligned}$$

Thus, with normalised eigenvalues (6) PC-entropy is defined as:

7 $$\begin{aligned} \text {PC-entropy}= -\frac{1}{\log _2(M)}\sum _i^M \bar{\lambda _i} \ \log _2(\bar{\lambda _i}). \end{aligned}$$

This entropy-based approach measures the breadth of the eigenvalue distribution and is proposed here as a synchronisation metric. Values range from 0 (when channels are maximally synchronised and a single eigenvalue captures all variance) to 1 (when channels are maximally desynchronised and variance distributed equally across all eigenvalues).

### The Kuramoto model

The Kuramoto model^25^ was used to validate PC-entropy as a synchronicity measure for EEG applications. This mathematical framework is widely used to analyse synchronisation phenomena in coupled oscillators and has found extensive applications in neuroscience for studying collective behaviour in biological and physical systems^26–29^. The Kuramoto model consists of *M* coupled oscillators, each with a phase $\theta _i$ and a natural frequency $\omega _i$. The system dynamics are governed by:

8 $$\begin{aligned} \frac{d\theta _i}{dt} = \omega _i + \frac{1}{N}\sum _{j=1}^{M}K_{i,j}\sin (\theta _j-\theta _i), \ \ \ i=1,\ldots , M \end{aligned}$$

where $K_{i,j}$ represents coupling strength from oscillator *j* to oscillator *i*. This interaction matrix can be decomposed as $K_{i,j}= g_{i,j} K$, where *K* is the coupling constant that controls global coherence between the oscillators.

Synchronisation occurs when oscillator phases become locked and oscillate at a common frequency. This transition from incoherence to coherence happens when coupling strength *K* exceeds a critical threshold. The degree of synchronisation is quantified using the order parameter, *R*:

$$\begin{aligned} R = \frac{1}{M}\left| \sum _{j=1}^{M}e^{i\theta _j}\right| , \end{aligned}$$

where $R=0$ indicates complete incoherence and $R=1$ represents perfect synchronisation^30^. This makes the Kuramoto model an ideal framework for studying relationships between interconnected oscillators and evaluating synchronicity measures beyond systems with explicit order parameters.

The system follows Eq. (8), with intrinsic frequencies $\omega _i$ drawn from a Gaussian distribution (mean $\bar{\omega }=1$, standard deviation $\Delta \omega =0.25$), with coupling weights also following Gaussian distributions. Numerical simulations were performed with *M* oscillators ($M=$20 and 30), varying the coupling constant *K* from 0. to 0.45 to progressively increase synchronisation. The initial conditions $\theta _i(t=0)$ follow Gaussian distribution (mean=0, standard deviation $\pi /2$). After numerical integration of Eq. (8), the PC-entropy was computed over the following synthetic signals $\sin (\theta _i(t))$ using time windows of varying length L at three sampling frequencies, $f_s$, with PC-entropy computed for each configuration. This procedure was repeated 50 times per configuration to establish statistical reliability.

### Surrogate signal

To evaluate the statistical significance of PC-entropy values, we compute the measure over an ensemble of phase-random surrogate signals , hereafter surrogate signals. These surrogate signals are synthetic signals that exactly preserve the Power Spectral Density (PSD) of the original signal whilst destroying all temporal relationships between channels, establishing the null hypothesis of no coupling^24^. For the surrogate signal to be real (not complex) in the time domain, its Fourier Transform must be conjugate symmetric. To generate random phases that satisfy this symmetry, random phases are generated only for the positive frequency spectrum (including the Nyquist point, if applicable), and then these phases are reflected and negated (their sign is changed) in the spectrum’s negative frequency portion. In this way, the preservation of the PSD and the fulfillment of the symmetry are ensured. This process establishes the distribution null of the metric, allowing for the calculation of statistical significance against thresholds like $\pm 2$ standard deviations.

### EEG signals

Beyond validation using the controlled Kuramoto model system, the proposed method was also evaluated on EEG recordings from publicly available PhysioNet databases^31^.

#### EEG during sleep

EEG recordings were obtained from the CAP Sleep Database^32^, comprising 108 polysomnographic recordings from the Sleep Disorders Center of Ospedale Maggiore of Parma^33^. From this database, we selected 4 controls (ID: n3, n5, n10, n11) and 10 subjects with nocturnal frontal lobe epilepsy (NFLE, ID: nfle1, nfle2, nfle3, nfle12, nfle13, nfle14, nfle15, nfle16, nfle17, nfle18). Selection criteria included consistent EEG channels, sampling frequency, and sufficient leads to assess inter-channel synchrony. The selected recordings all utilise 13 EEG channels distributed according to the 10-20 international system and sampled at 512 Hz with bipolar montage (Fp2-F4, F4-C4, C4-P4, P4-O2, F8-T4, T4-T6, FP1-F3, F3-C3, C3-P3, P3-O1, F7-T3, T3-T5, and C4-A1). These derivations were grouped in four regions: frontal = (Fp2-F4, FP1-F3, F8-T4, F7-T3, F4-C4, F3-C3), posterior = (C4-P4, C3-P3, P4-O2, P3-O1, T4-T6, T3-T5), right-hemisphere = (Fp2-F4, F8-T4, F4-C4, C4-P4, P4-O2, T4-T6), and left-hemisphere = (FP1-F3, F7-T3, F3-C3, C3-P3, P3-O1, T3-T5). These groupings were done to enhance spatial resolution and capture typical NFLE propagation patterns, where discharges originate focally in frontal regions before rapid bilateral spread and occasional posterior extension^34^. Polysomnographic recordings included expert-scored hypnograms from neurologists’ visual inspection of EEG frequency content and the muscle tone in electromyograms (EMG), classifying 30-s epochs into sleep stages; PC-entropy was computed on matching epochs for clinical consistency, aligning with prior analyses of this cohort^34^. For visualisation alongside PC-entropy values, sleep stages were assigned numerical values ordered by expected synchronisation level: wakefulness = 1, REM = 0.5, slow wave sleep (SWS) stage 1 = −1, SWS stage 2 = −2, SWS stage 3 = −3, and SWS stage 4 = −4. A 30-point moving average was applied to smooth short-term fluctuations and highlight longer-term trends in the PC-entropy time course.

To establish statistical significance, surrogate signals (as described above) were generated and analysed using the same 30-s epoch divisions with this procedure repeated ten times to determine mean and standard deviation. A 30-point moving average was applied to smooth short-term fluctuations and highlight longer-term trends in the PC-entropy time course.

For comparison between controls and NFLE patients, the PC-entropy was computed separately for five frequency bands (delta, theta, alpha, beta, and gamma) and the whole spectrum and considering four brain regions (frontal, posterior, right-hemisphere, left-hemisphere) plus whole-brain, yielding 30 synchrony-based features in total. All 30-second epochs were pooled by sleep stage and clinical condition. To identify brain regions and frequency bands that best discriminate between patients and controls, we employed volcano plots, which display both the magnitude of difference (Cohen’s d) and Mann-Whitney U-tests with Benjamini-Hochberg false discovering rate (FDR) correction, considering features significant at $|d| \ge 05$ and $q \le 10^{-5}$.

#### EEG from coma patients

EEG recordings were obtained from the I-CARE Database^35^, which contains 32,712 hours of data from 607 patients across 7 hospitals in the United States and Europe, available through PhysioNet. It includes continuous EEG recordings from adult patients with cardiac arrest who achieved return of spontaneous circulation after cardiopulmonary resuscitation but remained comatose (Glasgow coma score $\le$ 8)^36^. Long-term neurological function was assessed using the Cerebral Performance Category (CPC) scale^37^. The EEG signal comprised 19 channels recorded at 500 Hz using the 10-20 international system and stored as MATLAB MAT files. From this dataset, 47 patients with good outcome (CPC = 1) and 112 patients with bad outcome (CPC = 5) were selected. Inclusion criteria required that EEG recordings commenced within 6 hours of return of spontaneous circulation (ROSC) and continued for at least 19 hours post-ROSC. PC-entropy was computed across the entire EEG signal using 20-second time windows with a 5-sample moving average.

#### EEG during arithmetic task

An EEG dataset recorded during performance of a mental arithmetic task^38^ was used in this study. This dataset, publicly accessible on PhysioNet, comprises 36 EEGs from healthy university student volunteers^39^. EEGs were recorded at 500 Hz sampling frequency (band-pass filter 1-70 Hz) using a Neurocom monopolar EEG 23-channel system (Ukraine, XAI-MEDICA). All derivations were referenced to interconnected ear electrodes. Recording sites followed the International 10/20 system, including 19 electrodes: Fp1, Fp2, F3, F4, F7, F8, T3, T4, C3, C4, T5, T6, P3, P4, O1, O2, Fz, Cz, and Pz (excluding references A1 and A2).

In the original report^39^, subjects were classified into two groups according to their arithmetic task performance: poor counters (10 subjects) and good counters (26 subjects). PC-entropy was computed from 60-second EEG recordings during both rest and arithmetic task conditions. Prior to computation, EEG signals were decomposed into frequency bands (delta, theta, alpha, beta, and gamma) and analysed independently. PC-entropy was calculated using 10-second EEG segments with a 5-sample sliding window. Values obtained for each subject and condition were averaged. Box plots of PC-entropy values were generated for each band and condition. The Wilcoxon signed-rank test was used to compare rest and mathematics conditions separately in both groups, with FDR correction for multiple comparisons applied using a standard threshold of *q*-value $\le 0.05$.

## Results

### Evaluating the synchronisation of interacting Kuramoto oscillators with PC-entropy

To assess whether PC-entropy effectively evaluates synchrony between oscillating sources, we employed the well-studied Kuramoto model. Global synchronisation in this system is controlled by the coupling constant *K*, with synchronisation occurring when *K* exceeds a critical value, creating a transition from incoherent to coherent states. We considered systems with $M=30$ and 20 oscillators whose intrinsic frequencies and coupling weights follow Gaussian distributions.

**Fig. 1:**
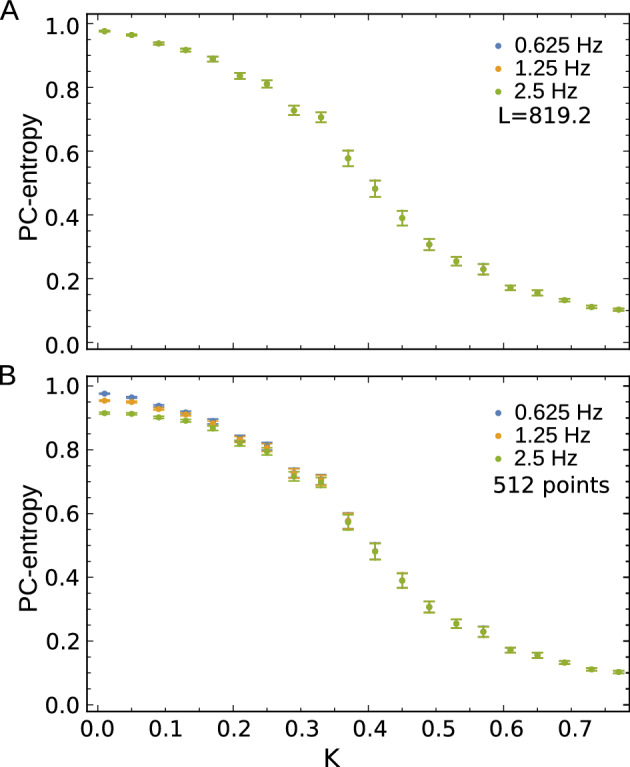
Mean PC-entropy values (Eq. 7) for Kuramoto systems with 30 oscillators as a function of the coupling constant *K*. (**A**) PC-entropy computed from time windows of $L=819.2$ seconds at three sampling rates $f_s$: 0.625 (512 sample points, blue symbols), 1.25 (1024 sample points, yellow symbols) and 2.5 Hz (2048 sample points, green symbols). (**B**) PC-entropy computed using identical sample points ($N=512$) but different sampling frequencies and window sizes. Error bars represent standard error of the mean across 50 runs.

Figure 1A shows mean PC-entropy as a function of coupling constant for sampling frequencies of 0.625 (blue), 1.25 (yellow), and 2.5 Hz (green) using fixed time windows ($L=819.2$ seconds). As expected, PC-entropy decreases monotonically with increasing synchronisation across all $f_s$ and *N* values, confirming its utility as a synchronisation measure. Sampling frequency had minimal impact on PC-entropy estimation, with different $f_s$ values producing nearly overlapping curves across all *K*-values. However, window size significantly influenced PC-entropy estimation at low synchronisation levels. Fig. 1B depicts results using identical sample points ($N=512$) but different window lengths *L*: 819.2 (0.625 Hz, blue), 409.6 (1.25 Hz, yellow), and 204.8 seconds (2.5 Hz, green). At low synchronisation, estimation depends on window size, with $L=204.8$ yielding 10% lower PC-entropy values than longer windows ($L=819.2$). This difference diminished at higher synchronisation levels. These findings indicate PC-entropy remains robust against reasonable parameter variations, with recommended values of $N=500\text {-}1000$ sample points and sampling frequency approximately twice the system’s highest frequency.

**Fig. 2:**
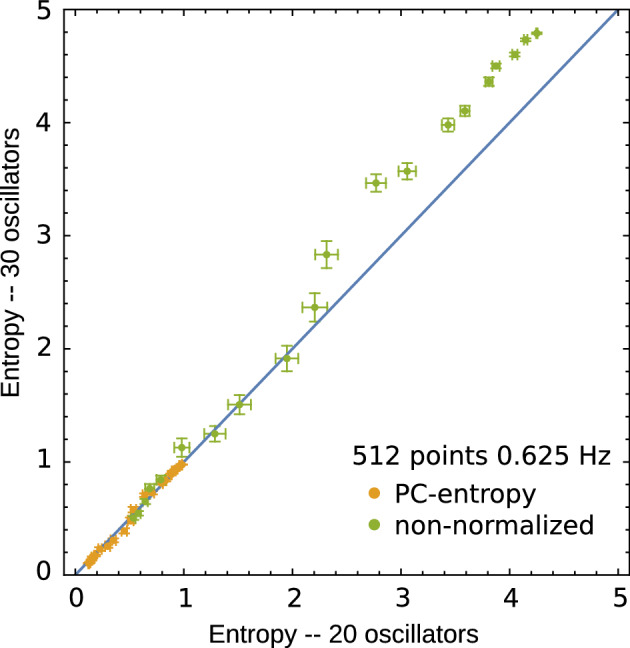
Entropy values for Kuramoto systems with 20 oscillators (horizontal axes) versus 30 oscillators (vertical axes) across different coupling values *K*. Yellow symbols correspond to PC-entropy whilst green symbols represent non-normalised entropy (Eq. 4) under identical conditions ($N=512$ and $f_s=0.625$). Error bars represent standard error of the mean across 50 run.

A crucial consideration for quantifying global synchronisation in EEG recordings is that the measure should be insensitive to the number of signal sources (channels), ensuring robustness and generalisability across different experimental setups. This property enables comparisons between EEG recordings with varying channel numbers. Channel number independence of PC-entropy (Eq. 7) was tested using the Kuramoto model. PC-entropy values computed from 20-oscillator systems closely matched those calculated from 30-oscillator systems under identical conditions (Fig. 2) when entropy is normalised by the $\log$ of the number of signal sources. In contrast, when entropy is not normalised (Eq. 5) by the $\log (M)$, systems with more signal sources have systematically more entropy than similar systems with fewer signal sources. This property ensures meaningful comparisons between EEG recordings with varying channel numbers, which is critical for clinical applications where electrode configurations may differ or when contact is lost in one or more channels during recording.

### PC-entropy applied to EEG during sleep

Polysomnographic EEG recordings were analysed using PC-entropy with two objectives: (i) characterising the time course of synchronisation during overnight sleep in healthy subjects, and (ii) comparing synchronisation patterns between healthy subjects and NFLE patients.

**Fig. 3:**
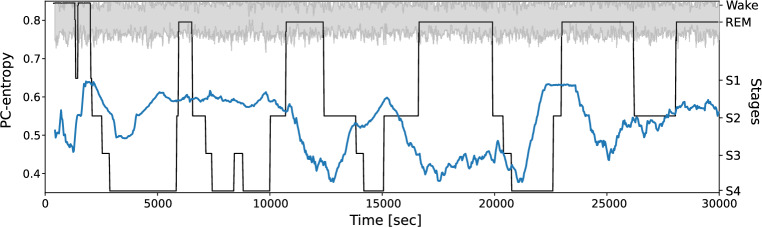
Time course of average PC-entropy from a control subject (subject ID: n11) during overnight sleep computed over the whole spectrum (blue line) and the numerical hypnogram representation (black line, wake = 1, REM = 0.5, S1 = −1, S2 = −2, S3 = −3, and S4 = −4). The gray band corresponds to the mean $\pm 2\sigma$ PC-entropy of surrogate signals (n=10). PC-entropy was computed over 30-second epochs using the whole signal without frequency band decomposition. Time courses of average PC-entropy computed for other control subjects are displayed in Supp. Fig. S1.

For the first objective, PC-entropy was computed across whole-band signals and within theta and alpha bands separately using all EEG derivations. The time course of average PC-entropy during overnight sleep for a healthy subject (ID:n11) revealed values consistently below the surrogate band (mean $\pm 2\sigma$, grey area), indicating significant synchronisation throughout the recording (Fig. 3). The PC-entropy time course exhibited global non-stationary behaviour with a broad range of variation, yet displayed piecewise stationarity characterised by domains of dynamic stability persisting for tens of minutes. These quasi-constant activity periods were separated by either abrupt or gradual state transitions. Although this temporal segmentation suggested sleep-related structure, the observed synchronisation patterns did not strictly correspond to traditional sleep stage classifications. This suggests that measured inter-channel synchronisation changes throughout the night evolved in a manner at least partially decoupled from the standard hypnogram classification. Similar beahavior was observed in the analysis of three additional subjects (n3, n5 and n10 Supp. Fig. S1). In support of this finding, the Spearman rank correlation between PC-entropy and the numerical hypnogram showed heterogeneous patterns across the different analysed subjects: n3 $\rho = 0.433$, $p < 10^{-46}$), n5 ($\rho = 0.307$, $p < 10^{-22}$), n10 ($\rho = 0.074$, $p = 0.03$ and n11 ($\rho = -0.186$, $p < 10^{-8}$). These variable correlations suggest that brain synchronisation reflects neural connectivity patterns that differ from expert-defined sleep stages, which are classified according to visual analysis of EEG waves in terms of amplitude and frequency, considering also muscular tone (EMG) and the presence of rapid eye movements (EOG).

**Fig. 4:**
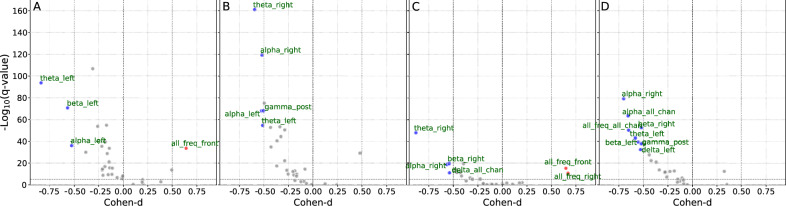
Volcano plot displaying PCE-based features (bands $\times$ brain regions) of NFLE patients against control subjects during different sleep stages: REM (**A**), S2 (**B**), S3 (**C**) and S4 (**D**). Significantly increased features are marked in red (Cohen-d $\ge 0.50$); while significantly decreased features are marked in blue (Cohen’s d $\le -0.50$). Significant features are labeled in green, indicating the band and brain region. The horizontal line corresponds to the FDR-corrected (Benjamini-Hochberg) $p-$value threshold ($q-$value=$10^{-5}$). The nonparametric Mann-Whitney U-test was used to statistically compare each feature.

For the second aim, PC-entropy-based synchrony measures were compared between healthy subjects ($n=4$) and NFLE patients ($n=10$). Volcano plots revealed distinct inter-channel synchronisation patterns across sleep stages (Fig. 4). During REM sleep, NFLE patients showed significantly increased synchronisation in theta, alpha, and beta bands in left derivations, whilst frontal activity was less synchronised compared to controls. Stage S2 demonstrated smaller effect sizes but greater statistical power due to more epochs available, with increased synchronisation in alpha and theta bands showing hemispheric separation. In S3, increased synchronisation in theta, alpha, and beta bands appeared in right derivations with reduced statistical power. Stage S4 revealed whole-brain increased synchronisation in NFLE patients, with a complex pattern of alpha and beta band synchronisation in right derivations alongside delta and theta synchronisation in left derivations. Mean, standard deviation, Cohen’s d and *p*-value for each synchrony-based feature used in the comparison between the two groups are listed in Supp. TableS1. These results suggest that PC-entropy effectively reveals underlying patterns of neural synchrony during sleep and provides biomarkers for discriminating between NFLE patients and healthy controls across multiple frequency bands and electrode configurations.

### PC-entropy applied to EEG from coma patients

**Fig. 5:**
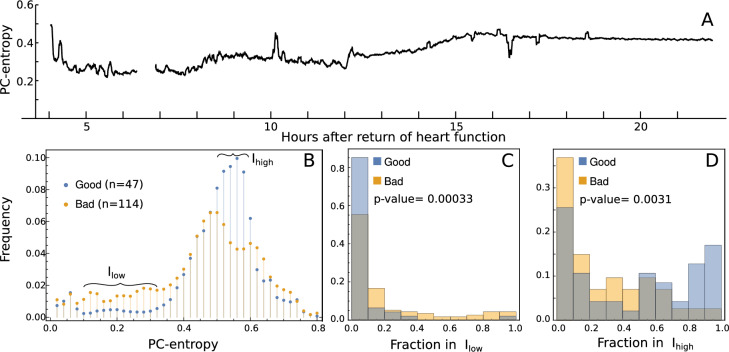
PC-entropy of EEG signals from coma patients with different outcomes. (**A**) Time course PC-entropy in a single coma patient with good outcome (subject ID: 0470) over 18 hours. PC-entropy was computed over 20-second time windows using the whole signal without frequency band decomposition. The small gap between 6h and 7h corresponds to a period with signal loss. (**B**) Histograms of PC-entropy computed over 1-hour epoch, 18 hours post-rhythm restoration in coma patients who had a good outcome (n=47, blue symbols) and a bad outcome (n=114, yellow symbols). The $\chi ^2$ test applied to the contingency table indicates that the two distributions are statistically significantly different (*p*-value $\le 10^{-300}$). We find two ranges of PC-entropy values, which are populated mainly by patients with a bad outcome (0.1 < PCE < 0.3, panel C) or patients with a good outcome (0.5 < PCE < 0.6, panel D). The Kolmogorov-Smirnov test indicates that the histograms are statistically significantly different.

We initially examined the temporal evolution of PC-entropy over an 18-hours period in a post-cardiac arrest coma patient with a favourable outcome (subject ID: 0470), with time origin marking cardiac rhythm recovery post-resuscitation; Fig. 5A illustrates a progressive increase. To assess prognostic relevance, PC-entropy was compared 18 hours post-rhythm restoration between good-outcome ($n=47$) and poor-outcomes ($n=114$) groups, revealing significantly higher values (less inter-channel synchronisation) in good-prognosis patients. A $\chi ^2$ test of independence applied to the contingency table of histogram frequencies, confirmed that the two distributions differed significantly (*p*-value $\le 10^{-300}$). Most patients with a poor prognosis had PC-entropy values between 0.1 and 0.3 (Fig. 5C), and Kolmogorov-Smirnov test indicates a significant difference between the distributions (*p*-value $= 3.3 \times 10^{-4}$). Conversely, most patients with a good prognosis had PC-entropy values between 0.5 and 0.6 (Fig. 5D), (*p*-value $= 3.1 \times 10^{-3}$). Together, these results suggest that the proposed inter-channel synchronisation measure may be a useful tool for assessing the neurological status of comatose patients.

### PC-entropy applied to EEG signals during arithmetic task

**Fig. 6:**
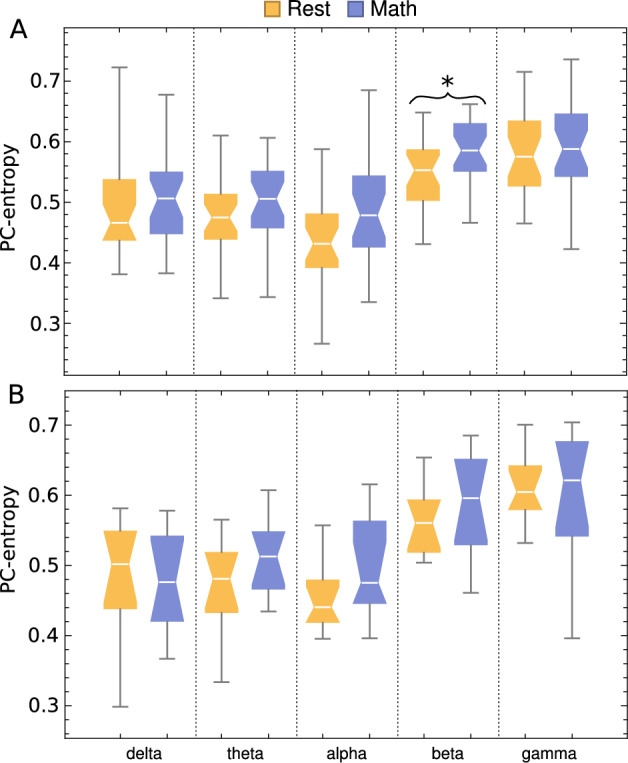
Box plots of PC-entropy during rest and arithmetic-task conditions for good counters (**A**) and poor counters (**B**). Analysis was performed separately for each frequency band using all 19 channels. $$* \rightarrow$$ *p*-value $$\le 0.01$$ and $$** \rightarrow$$ *p*-value $$\le 0.001$$.

**Fig. 7:**
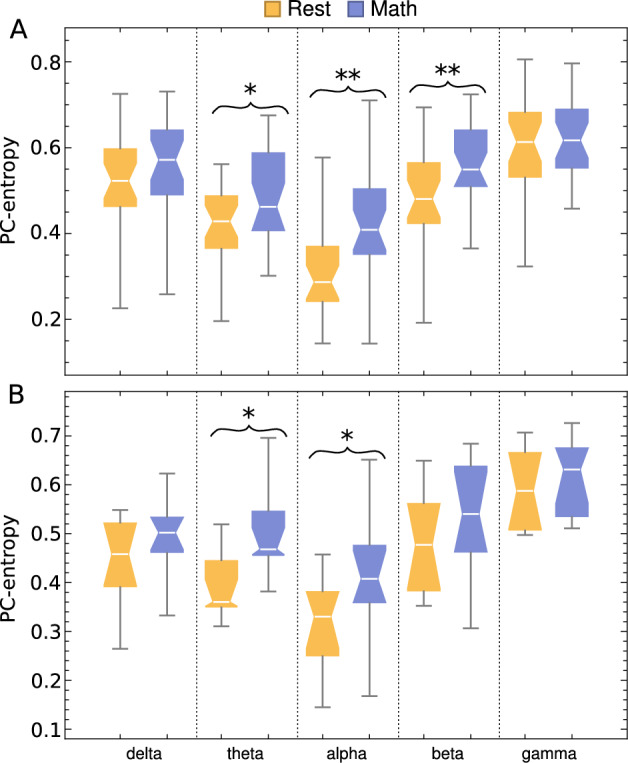
Box plot of PC-entropy during rest and arithmetic-task conditions for good counters (**A**) and poor counters (**B**). Analysis was performed separately for each frequency band using the 7 frontal channels (Fp1, Fp2, F7, F3, Fz, F4, F8). $$* \rightarrow$$ *p*-value $$\le 0.01$$ and $$** \rightarrow$$ *p*-value $$\le 0.001$$.

PC-entropy was significantly greater in the beta band for the good counters group during the arithmetic task compared to resting conditions, as shown in Fig. 6A. In contrast, no significant changes were observed in the poor counters group (Fig. 6B). Moreover, when analysis was restricted to the 7 frontal channels (Fp1, Fp2, F7, F3, Fz, F4, F8), good counters showed significantly greater PC-entropy during the arithmetic task in theta, alpha, and beta bands compared to resting conditions (Fig. 7A). The poor counters group demonstrated significant changes only in theta and alpha bands (Fig. 7B), with lower significance levels than the good counters group.

## Discussion

We demonstrate that PC-entropy provides a promising approach for evaluating global synchrony level in multichannel recordings. By directly assessing the eigenvalue distribution uniformity, this approach provides a single metric that summarises the synchrony level across all channels in an EEG segment, eliminating the need to integrate multiple pairwise channel combinations of pairwise methods^12–15^ Kuramoto model validation confirmed this full-spectrum sensitivity, revealing sigmoidal decay with coupling k, quasi-linear central regions, and saturation at extremes.

Most previously proposed synchrony measures operate between channel pairs^19^, though global extensions exist. Achermann *et al*. used Global Field Synchronisation, originally introduced in^5^, to map Fourier coefficients to complex plane clouds indicating phase synchronisation across derivations for sleep staging^6^. In addition, Barnes *et al*. recently developed global wavelet phase coherence via mean-squared magnitude of wavelet mean field^20^. However, both require exhaustive pairwise computations before averaging to obtain their global measure. The challenge of addressing globally coupled populations was recently tackled by Pikovsky and Rosenblum, who introduced an autocorrelation function integral parameter for globally coupled populations, distinguishing synchronous/asynchronous states^40^. However, this measure lacks electrophysiological validation for continuous dynamics.

PC-entropy provides a solution with strong temporal resolution (from 1-second windows) suitable for real-time clinical applications where computational speed is paramount. It captures synchrony patterns without requiring phase-locking analysis between signals and offers a complementary approach to existing methods for estimating similarity across EEG derivations. To demonstrate the utility of PC-entropy in clinical applications, we assessed its performance across three EEG datasets, as discussed herein.

### Sleep stages

PC-entropy revealed substantial temporal variations and temporal segmentation during sleep, i.e, time intervals characterised by dynamic stability lasting tens of minutes that suggests distinct functional regimes of brain synchronisation. However, the weak and heterogeneous Spearman correlations across subjects with traditional hypnograms indicate that PC-entropy captures a complementary dimension of brain dynamics beyond canonical sleep staging. This raises the question of what drives these synchronisation shifts. We hypothesise that they reflect inter-channel dynamic changes beyond individual frequency content, potentially involving large-scale functional network reconfigurations. Concurrent neuroimaging (fMRI) or event-related EEG analyses could map these neural substrates in future work.

Further, the PC-entropy approach has revealed significant discriminators between NFLE patients and controls across frequency bands, offering frequency-specific insights into network dynamics underlying sleep architecture. These findings underscore that sleep involves not only local oscillations but profound reconfigurations of large-scale functional connectivity and interregional synchronisation^41–43^. NFLE seizures predominantly occur in non-REM sleep, particularly during S2 with a smaller percentage in S3 and S4^44–48^. Ictal activity could explain the findings of elevated inter-channel synchronisation in patients’ beta, alpha and theta bands during non-REM sleep, which were right-lateralised in S2, left-lateralised in S3/S4, with bilateral posterior gamma in S2/S4. Intriguingly, REM differences persisted despite suppressed epileptic discharges, showing increased left-hemisphere theta, alpha and beta synchronisation linked to reduced frontal derivations across bands. This left-hemisphere lateralisation pattern raises questions about hemispheric dominance with most subjects expected to be right-handed, though handedness data were unavailable^49^. Moreover, without focal localisation details, some NFLE cases show bilateral frontal activity post-onset^47^ but definitive interpretation remains limited. Notably, the S2 synchronisation increase observed aligns with the consensus that NFLE seizures are caused by thalamocortical arousal distortions via K-complexes in the frontal cortex^47^, while REM alterations extend prior spectral findings of elevated delta/theta and reduced alpha^50^, confirming that NFLE dynamics transcend ictal episodes.

### Post-cardiac arrest comas

Coma patients typically exhibit EEG patterns dominated by slow delta rhythms, reflecting reduced brain activity and widespread cortical dysfunction. The emergence of faster rhythms replacing slow activity represents a positive prognostic indicator, alongside EEG reactivity to stimuli. More severe cases exhibit flat EEG patterns and burst-suppression activity^51^. In the analysis presented in Fig. 7, PC-entropy increases in patients that experienced favourable evolution after cardiac resuscitation with only minor sequelae. This decreases in the synchronisation reveals a greater inter-channel differentiation, potentially indicating restored brain activity. EEG complexity is recognised as a general indicator of mental activity and consciousness^52^. Our analysis suggest that PC-entropy’s utility in detecting these prognostic shifts, which preceded consciousness recovery in this case.

### Cognitive tasks

During serial subtraction tasks, PC-entropy computed across all channels showed increased values in the beta band only for participants with good proficiency levels. When analysis was restricted to frontal channels, differences emerged in theta and alpha bands for both good and poor counters, with higher PC-entropy during task performance. Beta band increases in frontal regions were significant only in good counters. These results reveal desynchronisation between EEG channels during task execution, which is expected when brain regions activate and neural networks depart from the resting state^53^. The finding that beta band frontal activation was significant only in higher proficiency subjects aligns with fMRI studies demonstrating substantial frontal lobe involvement in arithmetic calculations^54^. This supports the value of PC-entropy analysis for studying brain activation patterns.

### Limitations

In this study, we demonstrated the potential of PC-entropy by confirming inter-channel synchrony changes that are expected in specific physiological and pathological contexts. Whilst our method offers several key advantages over existing approaches, several limitations should be acknowledged. PC-entropy evaluates inter-channel (long-range) synchrony across brain regions, distinct from local desynchronisation within single derivations. In individual channels, high-amplitude low-frequency traces indicate idling networks, while fast low-amplitude activity signals functional activation^55^. Local desynchronisation thus reflects task-related activation, contrasting with synchronised oscillations of resting states. Despite these differing spatial scales, mental activation during tasks may still produce inter-channel differences manifesting as long-range desynchronisation. Homogeneous traces across channels suggest global idling, while activation creates measurable differentiation quantified by PC-entropy.

EEG volume conduction presents a fundamental challenge, as field spread from each neural source simultaneously influences multiple sensors, introducing spurious linear correlations that may inflate PC-entropy estimates of inter-channel dependencies^17,18^. These artefacts could confound the observed coma prognostic separation and NFLE discrimination, as volume-conducted signals can mimic true global synchrony. Without ground truth source localisation, demonstrations relied on expected physiological and pathological synchrony patterns across datasets. Future studies could incorporate source localisation techniques or high-density montages to disentangle true connectivity from field effects. Further validation across diverse EEG acquisition systems and clinical populations including larger sample sizes would strengthen the method’s generalisability.

Additionally, PC-entropy requires adequate sample sizes (typically 500-5000 points) for reliable estimation of principal components, and assumes linear relationships between channels through PCA. The latter means that non-linear dependencies between channels could be underestimated by PC-entropy. This limitation can be overcome with the wavelet-based approach^20^ which can also detect cross-frequency coupling^56,57^.

## Conclusion

PC-entropy provides a single metric that quantifies synchrony across channels in EEG segments, eliminating the complexity of analysing multiple channel pairs individually. Analysis can be performed within specific frequency bands through signal pre-filtering, facilitating interpretation based on established knowledge of each band’s physiological role. Through analysis of diverse EEG datasets, we have demonstrated PC-entropy’s potential as a global synchrony measure applicable across different behavioural paradigms and temporal scales.

## Supplementary Information

Supplementary Information 1.

Supplementary Information 2.

## Data Availability

The datasets analysed during the current study and scripts used are available in the Zenodo repository, https://zenodo.org/records/16850208.
