# Supplementary material for: A principal component entropy metric for assessing global synchronicity in EEG signals
Source: Sci Rep. 2026 Mar 3;16:8031. doi: 10.1038/s41598-026-36434-0 (PMC12957513; doi:10.1038/s41598-026-36434-0)
Supplement: Supplementary file 1 — Supplementary Information 1. [file 41598_2026_36434_MOESM1_ESM.pdf]

A principal component entropy metric for assessing global synchronicity in EEG signals

Luis Diambra, Anna Hutber, Zakarriah Drakeford-Hafeez, Ran Mi, Vasiliki Tsirka and Alberto Capurro

Supplementary Figures

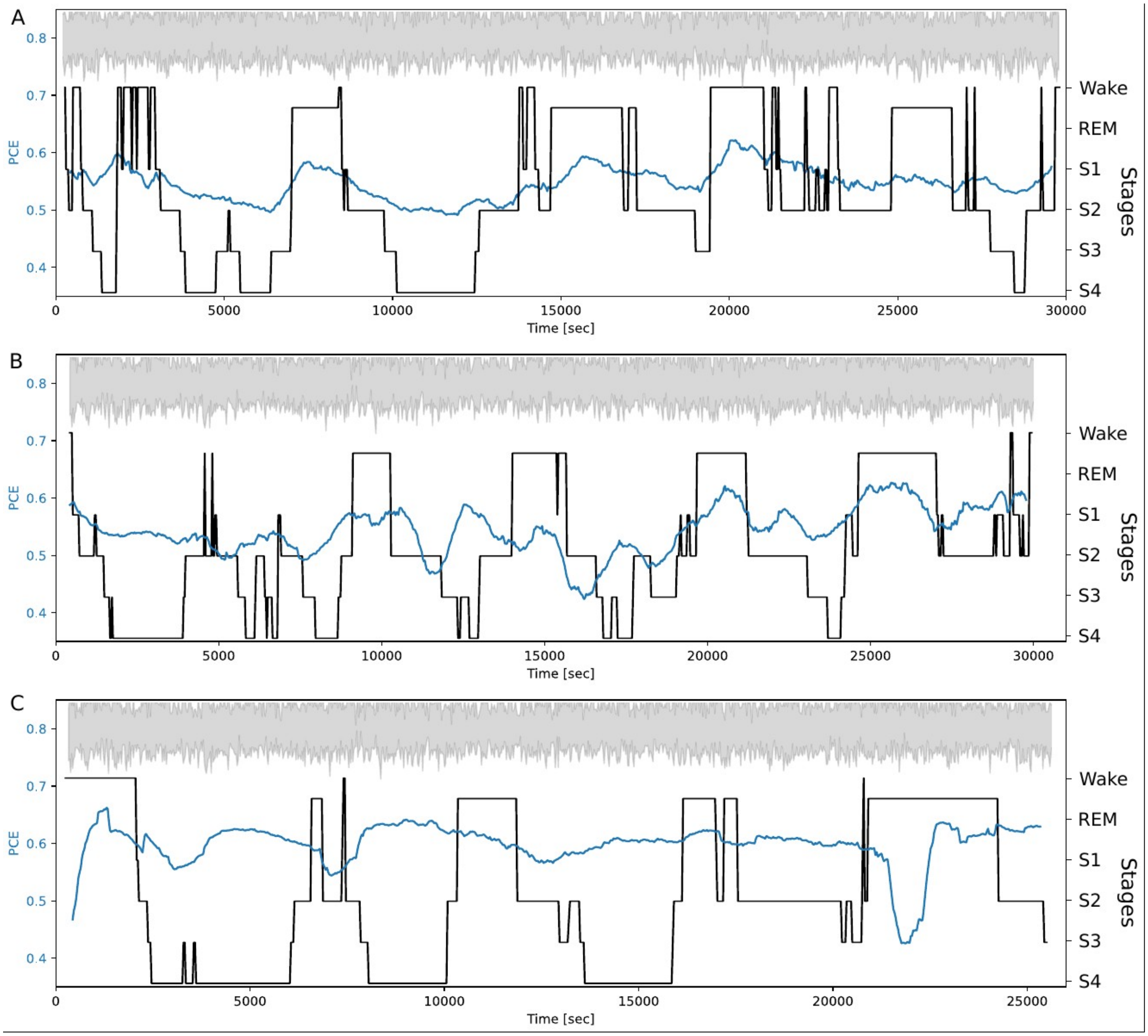

Supp Fig. S1: Time course of average PC-entropy from three control subjects during overnight sleep computed over the whole spectrum (blue line) and the numerical hypnogram representation (black line, wake = 1, REM = 0.5, S1 = -1, S2 = -2, S3 = -3, and S4 = -4). Subject ID: n3 (panel A), subject ID: n5 (panel B) and subject ID: n10 (panel C). The gray band corresponds to the mean ±2σ PC-entropy of surrogate signals (n=10). PC-entropy was computed over 30-second epochs using the whole signal without frequency band decomposition.

Captions of Supplementary Tables

Supp. Table S1: Excel file contains the comparison of 30 PCE-entropy-based features among the groups of NFLE patients and control subjects. The first column corresponds to the name of the PCE-entropy-based feature; n1 and n2 are the numbers of epochs from control and NFLE, respectively, used in the comparison. Mean and std correspond to the mean value and standard deviation. Log2 (FC) is the base-2 logarithm of the fold change of means with respect to the control group. The last three columns correspond to Cohen's d, the p-value, and the adjusted p-value (q-value) for multiple comparisons (Benjamini-Hochberg). The same table structure was used for the comparison in each sleep stage.
